# The subcutaneous implantable cardioverter-defibrillator should be reserved for niche indications

**DOI:** 10.1016/j.hroo.2022.09.005

**Published:** 2022-10-21

**Authors:** Yang Yang, Ulrika Birgersdotter-Green

**Affiliations:** Department of Cardiac Electrophysiology, University of California San Diego, San Diego, California

**Keywords:** Antitachycardia pacing, Implantable cardioverter-defibrillator, Inappropriate shocks, Subcutaneous implantable cardioverter-defibrillator, Sudden cardiac death

**Key Findings**

▪ The subcutaneous implantable cardioverter-defibrillator (S-ICD) is a completely extrathoracic device that is a great treatment option for patients with limited vascular access, complex anatomy, or previous infection.
▪ Significant tradeoffs due to the extravascular design of the S-ICD limit its effectiveness in arrhythmia detection and lead to a higher rate of inappropriate shocks despite use of algorithms to filter cardiac and extracardiac signals.
▪ The absence of endocardial pacing in the S-ICD removes the option for antitachycardia pacing, atrial pacing, cardiac resynchronization therapy, and conduction system pacing, which all are crucial components of device therapy.
▪ The extrathoracic locations of both the shock coil and the generator lead to unique challenges to defibrillation with the S-ICD, with uncertain long-term defibrillation thresholds.

Since the introduction of the first implantable cardioverter-defibrillator (ICD) in 1980, ICDs have undergone significant improvements to become the pillar of sudden death prevention today. The first device consisted of a 250-g abdominal generator with a surgically applied pericardial patch.^1^ Over time, the generator decreased to one-fifth of its original size, and the pericardial patch was replaced by transvenous leads with integrated defibrillation coils. However, for patients with impaired venous access or children with congenital heart disease, epicardial systems remained their only option until the development of the subcutaneous implantable cardioverter-defibrillator (S-ICD).

The S-ICD borrows many design elements from the transvenous system and is composed of an extrathoracic lead and pulse generator. Its subcutaneous leads were touted to have increased longevity and decreased infectious risk. Whereas lead-related infective endocarditis has been reported in 22% of all ICD-related infections, the rate of systemic infection with the S-ICD is extremely low and has only been described in case reports.^2^^,^^3^

As the S-ICD passes its first decade in clinical practice, many electrophysiologists have started to ask whether the S-ICD should be considered as a first option for all patients. Two recently published randomized trials (PRAETORIAN [Prospective Randomized Comparison of Subcutaneous and Transvenous Implantable Cardioverter Defibrillator Therapy] and ATLAS [Avoid Transvenous Leads in Appropriate Subjects]) compared the S-ICD to the TV-ICD head to head.^4^^,^^5^

The subcutaneous design comes with tradeoffs that cannot be ignored (Table 1). Because of the lack of endocardial pacing components, patients with long-term pacing needs are ineligible for the S-ICD.^6^ The reliance on far-field sensing hampers appropriate arrhythmia detection. The extrathoracic position increases defibrillation thresholds (DFTs),^7^^,^^8^ requiring a larger battery in the S-ICD capable of delivering an 80-J charge and limiting the lifespan of the device. All these factors relegate the S-ICD to the realm of a niche device.

**Table 1:** Advantages and disadvantages of S-ICD implantation

Advantages	Disadvantages
Able to implant in patients with congenital heart disease	Reliance on far-field sensing leads to issues separating QRS complex from cardiac and noncardiac signals
Able to implant in patients who lack vascular access	Lack of endocardial pacing leads to inability to deliver ATP, atrial pacing, CRT, or conduction system pacing
Lack of transvenous leads lowers risk of infection and thrombosis for patients with risk of infection or thrombosis	All extrathoracic components require careful implant technique to avoid adipose tissue acting as insulator for defibrillation

ATP = antitachycardia pacing; CRT = cardiac resynchronization therapy; S-ICD = subcutaneous implantable cardioverter-defibrillator.

### Sensing limitations

With the absence of any endocardial components, the S-ICD must solely rely on subcutaneous electrocardiography for arrhythmia detection. Sensing electrodes on distal and proximal portions of the subcutaneous lead and the generator constitute the 3 possible poles for the sensing vectors. All 3 vectors span a large portion of the precordium and provide far-field sensing not only of the QRS complex but also of the P and T waves as a result of the larger size of the antenna. Therefore, algorithms for arrhythmia detection in the S-ICD have the unique challenge of separating the QRS complex from other cardiac and extracardiac signals before other criteria can be applied.

For optimization of signal-to-noise ratios, all S-ICD candidates must be screened before implantation. Any vector with a small QRS–to–T-wave ratio is rejected because of concern for T-wave oversensing. In addition, vectors with absolute QRS amplitudes above or below the threshold are rejected. Up to 10% of patients can fail this screening process in all 3 vectors, and up to 37% of passing vectors may fail upon rescreening.^9^^,^^10^ This severely restricts the eligible patient population. Furthermore, the proportion shrinks dramatically when looking at patients who benefit the most from an extravascular device. Young patients with channelopathies and inherited cardiomyopathies often have abnormal repolarization with exaggerated T-wave amplitudes. In patients with arrhythmogenic right ventricular cardiomyopathy, up to 37% failed S-ICD screening; and in patients with hypertrophic cardiomyopathy, 48% failed screening in ≥2 vectors.^11^

Passing the screening process does not prevent sensing issues postimplantation. Far-field sensing issues with the S-ICD led to a higher rate of inappropriate shocks (IAS) in the initial S-ICD compared to TV-ICDs, with T-wave oversensing being the major cause. Although T waves may pass screening during sinus rhythm, with conduction abnormalities, frequent premature ventricular contractions, or slow ventricular tachycardia (VT), abnormal repolarization can lead to amplified T waves and oversensing. Similarly, conduction system abnormalities can prolong the QRS complex and lead to double counting.^12^ In rare cases, atrial hypertrophy has also led to large p waves being miscounted.^13^ Common issues with S-ICD sensing are shown in Figure 1.

**Figure 1:**
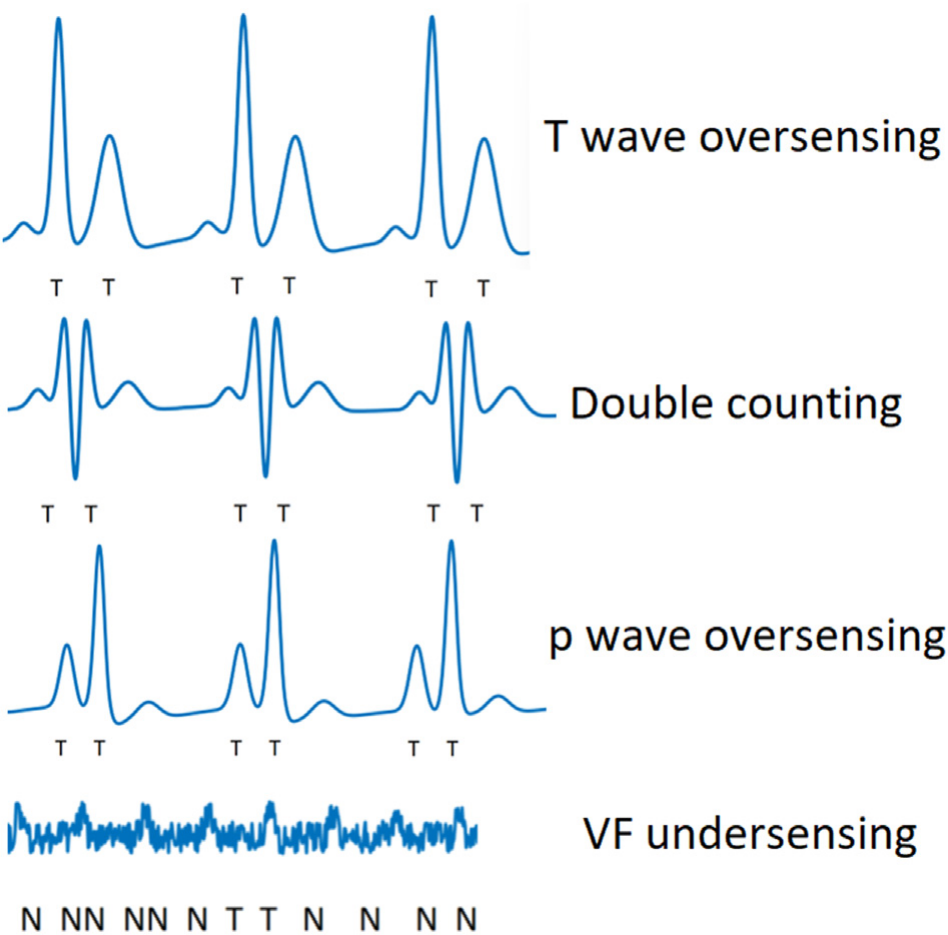
Sensing issues with the subcutaneous implantable cardioverter-defibrillator (S-ICD). Despite the use of appropriate algorithms and preimplant screening, inappropriate arrhythmia detection can occur with subcutaneous electrocardiograms used by the S-ICD. Common issues include T-wave oversensing, R-wave double counting, p-wave oversensing, and ventricular fibrillation (VF) undersensing.

With the longer antenna used for subcutaneous electrocardiography, electromagnetic interference becomes more prominent. Air entrapped in the generator pocket can mimic ventricular fibrillation (VF), with use of a transcutaneous electrical nerve stimulation unit responsible for 25% of IAS in the ATLAS trial.^5^^,^^14^ The S-ICD attempts to exclude extracardiac signals using an algorithm based on frequency and slew rate analysis. However, discounting low-amplitude, high-frequency signals may delay or inappropriately withhold therapies in VF. Up to 14% of patients at the time of DFT testing had significant delays to time of therapy, and up to 4% had noise oversensing leading to absence of appropriate VF detection.^15^

Valiant attempts have been made to accurately identify arrhythmias through the SMART Pass algorithm,^16^ but it is not a cure-all. Even with appropriate programming, S-ICD sensing still falls short. In the latest clinical trials comparing S-ICD vs TV-ICD, there still is an alarming trend toward higher IAS despite the widespread use of SMART Pass. Patients with S-ICDs had 6.4% IAS compared to 2.8% in the TV-ICD group (odds ratio 2.38; 95% confidence interval 0.96–5.90) during mean 2.5 years of follow-up in the ATLAS trial.^5^ Although failing to reach statistical significance, the trend of higher IAS with the S-ICD is supported by a multitude of real-world registries. An Italian S-ICD registry reported an IAS rate of 9.4% in 2 years, similar to rates reported worldwide in the EFFORTLESS (Evaluation of Factors Impacting Clinical Outcome and Cost Effectiveness Of the S-ICD) trial (11.7% in 3 years).^17^^,^^18^ Given that IAS are psychologically harmful and lead to increased health care utilization, the S-ICD cannot be considered as a first option until any issues are addressed.^19^

### Lack of pacing

Although the S-ICD is capable of emergency postshock pacing, it cannot provide reliable pacing due to the lack of endocardial components. Current guidelines are clear that only patients without pacing needs are eligible for the S-ICD. It is worthwhile to highlight the importance of cardiac resynchronization therapy (CRT) with many of our primary prevention patients. For those with chronic kidney disease on dialysis (a population with poor vascular access, higher rates of infection, and seemingly great candidates for the S-ICD), primary prevention ICD alone does not seem to confer mortality benefit.^20^^,^^21^ Those patients do seem to benefit from CRT-defibrillator compared with ICD alone.^22^

The lack of antitachycardia pacing (ATP) leaves a gaping hole in the electrophysiologist’s arsenal and severely limits widespread adoption of the S-ICD. In PRAETORIAN, 50% of all VT episodes in TV-ICD patients were terminated successfully with ATP. In other real-world registries, ATP can terminate up to 87% of VTs <200 bpm and 62% of fast VTs between 200 and 250 bpm.^23^ ICD shocks undoubtedly are effective in terminating life-threatening arrhythmias, but we know from decades of experience that ATP can provide a painless and trauma-free option to terminate arrhythmias. To take away that option would be very difficult.

Even if we were to make that decision, experience tells us a significant portion of patients who qualify for an ICD without pacing needs eventually will develop such needs. Trials of primary prevention ICD patients show 10% will develop pacing needs in 5 years.^24^ Contemporary observational studies hint this number may be even higher in the short term. In an observational study of patients with a single-chamber ICD over 2 years, 6.7% required >5% pacing when programmed at VVI 40 bpm.^25^ Unfortunate patients who undergo S-ICD implantation and then develop pacing needs must undergo an additional procedure with either a transvenous pacemaker or a leadless pacemaker, thus incurring the cost and risk of an additional procedure. If a transvenous pacing system is implanted, all advantages of an extravascular system are lost. Trials are underway for a seemingly elegant solution to the problem—implantation of a leadless pacing system capable of communicating with the S-ICD to deliver ATP. However, the proposed solution still would not be able to provide atrial pacing, CRT, or conduction system pacing. It also comes with the additional cost and complexity of 2 independent devices requiring 2 separate procedures at the time of replacement.

### Uncertainty with defibrillation success

Successful defibrillation therapy requires the maximum amount of current to cross the myocardium. The positions of the shock coil and generator, as well as their surrounding tissues, can drastically affect the flow of current between them and are crucial to terminating VT/VF.^26^

In the TV-ICD, the shock coil is embedded in the right ventricle and is surrounded by either myocardium or blood, both low-impedance tissues. In the S-ICD, however, the shock coil sits parasternally and can be in contact with either muscle (low impedance) or adipose tissue (high impedance). Any adipose tissue around the coil would dramatically increase DFTs. In addition, because the S-ICD shock coil sits in an extrathoracic anterior position, any anterior shift of the generator past the midline would dramatically reduce the amount of myocardium crossed by the shock vector. Therefore, minimizing adipose tissue between the lead and sternum, as well as ensuring a posterior location of the generator, are crucial predictors of successful defibrillation.^27^ Not surprisingly, obese patients pose significant challenges for S-ICD implantation, with increased body mass index being a good predictor of DFT failure at the time of implantation.^28^ In the United States, where the local prevalence of obesity can be >40%, the challenges are clear. Additionally, DFTs may not be stable over time, as 20% of patients fail repeat DFT testing at the time of S-ICD generator change.^29^

### Improvements in TV-ICDs

Along with the technical innovations of the S-ICD, TV-ICDs have continued to improve with regard to battery and lead longevity. Previous-generation lithium silver vanadium oxide batteries had nonlinear discharge curves and variable internal cell impedances leading to unpredictable replacement intervals and variable capacitor charge times. Newer models with manganese dioxide can maintain a high voltage over longer periods of time and stable internal impedances. This allows new devices to use 90% of capacity rather than 70% in the older models. Improved battery technology has increased the average service life of TV-ICDs significantly.^30^^,^^31^ The longer battery life of single-chamber TV-ICDs (average 8 years) compared with S-ICDs (average 5 years) means fewer procedures over the lifetime of the patient, thus reducing costs and procedure-related morbidity.^30^^,^^32^

Historically, annual failure rates for defibrillation leads are estimated to be 2.7% per year, reaching about 20% in 10-year-old leads.^33^ This includes notable failures such as the Medtronic Fidelis and St. Jude Medical Riata leads, with failure rates of 4.8% per year and their subsequent recalls.^34^ Lessons from these failures have led to improvements in lead longevity. Short- and medium-term follow-up studies since the recalls have shown improved survival of defibrillation leads.^35^^,^^36^ In comparison, the first-generation S-ICD leads underwent a U.S. Food & Drug Administration class I recall due to unexpected lead fracture, with an estimated 0.2% annual estimated failure rate. One meta-analysis showed that first-generation S-ICD leads are no more durable than contemporary TV-ICD leads.^35^^,^^37^

### Increased costs

Although the S-ICD has been commercially available for more than a decade, costs in some locales still pale in comparison to the costs of TV-ICDs. In Europe, the S-ICD can cost between 3 to 7 times more than a single-chamber TV-ICD.^38^^,^^39^ This combined with a notably shorter battery life (5 years for the S-ICD vs 8 years for the TV-ICD) can lead to dramatically higher lifetime costs for an S-ICD implant. The proposed leadless pacing component for delivery of ATP would be another costly addition to an already expensive system.

## Conclusion

Although the S-ICD is a valuable tool, we must recognize its limitations and use it appropriately. Just as the first ICD in 1980 was a revolutionary device with many limitations, the S-ICD certainly has room for improvement. The lack of permanent pacing for ATP, atrial pacing, CRT, and conduction system pacing; the difficulties with arrhythmia detection with far-field sensing; as well as the uncertainty of DFTs over time are problems with the S-ICD that must be solved before the device can be widely adopted. The combination of a leadless pacing system in communication with the S-ICD is undergoing clinical trials that would address concerns about the lack of ATP and bradycardia support. Meanwhile, efforts are being made to identify patients at risk for high DFTs. We look forward to the day when subcutaneous ICD systems can become the first option for our patients, but we are not there yet.
